# Adsorption of Transition Metal Cations on B75 Bentonite and Behavior of the Glass/Copper/Bentonite Barrier System under Simulated Repository Conditions

**DOI:** 10.1021/acsomega.4c11699

**Published:** 2025-02-18

**Authors:** Margit Fabian, Emese Varga, Istvan Tolnai, Janos Osan

**Affiliations:** HUN-REN Centre for Energy Research, Environmental Physics Department, Konkoly Thege Miklos st 29–33, Budapest 1121, Hungary

## Abstract

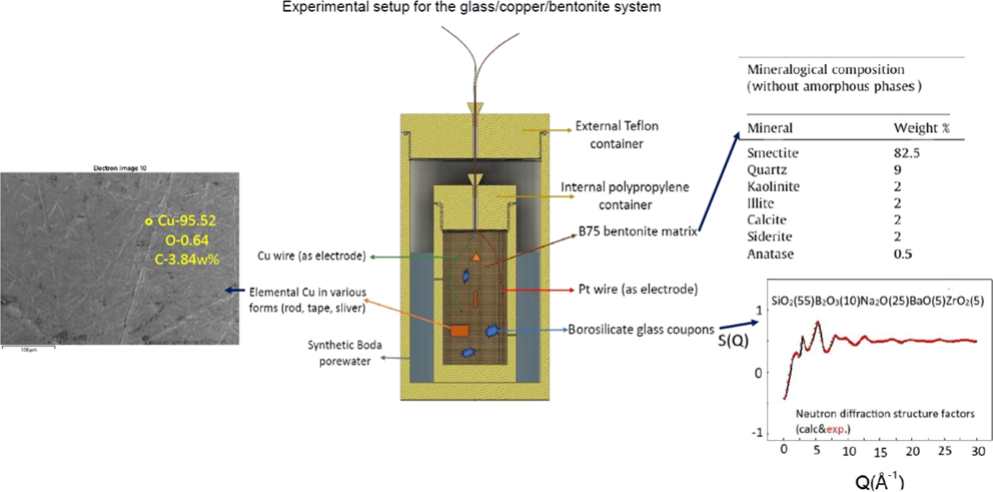

Since plans for a future deep geological repository (DGR) site have been published in Hungary, further study of their material characteristics has become essential to understand how to operate and design them properly fully. The main goal of this study is to understand the retention of cations (Ni(II) and Co(II)) by the B75 bentonite through adsorption and desorption experiments and the interactions between materials of the glass/copper/bentonite model system under repository conditions. The obtained adsorption and desorption isotherms show irreversible sorption of both ions at higher concentrations (above 10^–5^ M), where the difference is higher in the case of the Ni(II) ions than for Co(II) ions. The surface study of the borosilicate glass shows an increasing trend in the concentration of Zr and O, and a decrease in the case of Si, Ba, Na, and B, showing an equilibrium state from the beginning. The surface of different physical shapes of copper (rod, tape, sliver) shows differences in the case of the rod; higher C and lower O amounts were found, which indicates the formation of Cu_2_O; however, the difference between the samples was not influential. Based on the inductively coupled plasma-optical emission spectrometry, the concentration of elements in the pore water solutions was determined. The release of B, K, Na, and Si increased throughout the experiment; in contrast, the concentrations of divalent cations (Ca and Mg) decreased throughout the measurement. No copper dissolution was detected.

## 1 Introduction

Approximately 5% of the global annual electricity demand is met by nuclear power generation.^1^ Although high-level radioactive waste (HLW) is produced in the smallest quantities during the nuclear fuel cycle, its safe management and disposal are crucial. Currently, HLW including spent nuclear fuel, reprocessing waste, and decommissioning waste is stored in temporary surface repositories. Deep geological repositories (DGR) are being considered for final disposal in several European countries, including Hungary. The concept of DGRs is to prevent the release of radionuclides into the biosphere through multiple engineered barriers and a natural barrier during long-term storage. Key parts of the engineered barrier system are the waste form itself, the corrosion-resistant and mechanically stable containers, the buffer material surrounding the waste packages, and the backfill material. Finally, the appropriate host rock acts as a natural barrier. For HLW produced after reprocessing, vitrification is considered to prepare a suitable waste form. To better withstand the heat generated by the HLW, the container is made from different materials depending on the storage conception, e.g., carbon steel, copper, or different alloys. Nowadays, the most promising material is high-purity copper. Previously published data suggest that the resistance properties of copper will be adequate for this purpose.^2−5^ Clay-rich rocks are considered excellent natural barriers and buffer materials due to their high water absorption and cation exchange capacity. A large group of them are bentonites, which are montmorillonite-based clay systems and are proven to be suitable buffer and backfill materials due to their excellent cation retention characteristics.^6,7^ In a DGR, the drifts are backfilled with bentonite. After they have been closed, the bentonite slowly saturates with pore water, causing it to swell and thus form a tight and practically impermeable homogeneous mass. In Hungary, the Boda Claystone Formation (BCF) is the promising host rock for establishing the DGR. The Public Limited Company for Radioactive Waste Management (PURAM) has identified Czech bentonite type B75 as a potential buffer and backfill material.

B75 bentonite has been extensively studied: several studies have been focused on the interaction with different radionuclides^8,9^ the transport processes in its structure,^10^ and the long-term behavior in repository conditions.^11^ However, B75 bentonite has not yet been studied specifically for transition metals such as nickel and cobalt as corrosion products in HLW.

Experiments on vitrified waste forms have crucial importance since the glass containing the radionuclides could contact the bentonite in the unfavorable event of damage to the copper container. This could occur due to external mechanical or chemical interactions or thinning of the container due to corrosion over a long period.

The present work aims to study (i) the retention of Ni(II) and Co(II) by the B75 bentonite through adsorption and desorption experiments and (ii) the interactions between materials of the glass/copper/bentonite model system under repository conditions.

Adsorption and desorption isotherms for Ni(II) and Co(II) were experimentally determined using powdered B75 bentonite dispersed in pore water characteristic for BCF. The glass/copper/bentonite model system comprises borosilicate glass characteristic for the waste matrix and different physical shapes of copper (rod, tape, and sliver) embedded in bentonite and saturated in BCF pore water. After keeping the system at elevated temperatures, corrosion of borosilicate glass and copper in bentonite was studied by postmortem investigations. The structural and compositional changes on their surfaces were measured and compared, thus providing a more complete insight into the planned Hungarian repository.

## 2 Materials and Methods

### 2.1 Materials

#### 2.1.1 B75 Bentonite

This study used the commercially compacted Czech bentonite B75 (KERAMOST Ltd.) extracted from the Cerny vrch deposit. It is a calcium–magnesium bentonite (but in many cases, it also occurs as sodium-bentonite) with a montmorillonite content varying between 60 and 85%. Bentonite was formed through the chemical transformation of volcanic ash, during which the original minerals were altered into clay.

#### 2.1.2 Synthetic Boda Pore Water (SBPW)

The extraction and analysis of BCF pore water is very complex due to the very low porosity and nanoscale pore size distribution of the rock. The initial BCF pore water considered in the experiment was a modeled synthetic pore water,^12,13^ which closely matches the composition of the BCF pore water extracted by a modified cryodesiccation (LN2 freezing).^14^ The Boda pore water chemistry was calculated using the geochemical speciation code MINSORB and the Nagra/PSI 01/01 thermodynamic database.^15^ The chemical composition of SBPW is shown in Table 1. To buffer the pH of the SBPW, TRIS (tris(hydroxymethyl)aminomethane) was used at a concentration of 2 × 10^–3^ M.

**Table 1:** Chemical Composition of SBPW

element, ion	concentration [mol/L]
Na	1.7 × 10^–2^
Sr	1.5 × 10^–5^
K	1.8 × 10^–4^
Mg	2.3 × 10^–3^
Ca	3.1 × 10^–3^
Cl^–^	2.3 × 10^–2^
SO_4_^2–^	1.9 × 10^–3^
HCO_3_^–^/CO_3_^2–^	6.1 × 10^–4^
ionic strength	3.3 × 10^–2^
pH	8.1

#### 2.1.3 Copper

To model the reaction on the surface of copper as a potential container material, three physical shapes of copper were used with high quality: Cu rod (*d* = 2 mm, 99.99%), Cu tape (99.95%), and Cu sliver (99.9%), all purchased from Avantor (VWR International Kft).

#### 2.1.4 Borosilicate Glass Composition

The borosilicate glass used in the experiment had the following composition:^16−18^ 55 mol % SiO_2_–10 mol % B_2_O_3_–25 mol % Na_2_O–5 mol % BaO–5 mol % ZrO_2_. The density of the bulk glass was 2.67 g/cm^3^.

### 2.2 Adsorption–Desorption Experiments

#### 2.2.1 Conditioning of B75 Bentonite

The B75 bentonite was conditioned with synthetic Boda pore water. Dry crushed bentonite (<100 μm) was saturated in the pore water, and the suspension was shaken with an orbital shaker. The concentrations of ions during the conditioning were monitored with ion chromatography (IC). During the conditioning process, approximately 5 g bentonite was saturated in 30–40 mL synthetic pore water at pH = 8.0 ± 0.1, for 4 h, then it was centrifuged at 3000 rpm for 5 min and the supernatant was replaced with pure SBPW. The process was repeated 4 times; this allowed for the chemical equilibration of the clay deposit with the geochemical environment.

#### 2.2.2 Adsorption and Desorption Isotherms

In the sorption experiments, solutions with nickel/cobalt concentrations between 10^–6^ and 3 × 10^–3^ M were prepared in duplicate and shaken in 50 mL shaker vessels for 28 days. The pH was adjusted to 8.0 ± 0.1 in all of the solutions. The liquid-to-solid ratio was 1000 mL/g, and 20 mg of conditioned bentonite was added to 20 mL of liquid. Samples were taken to centrifugation (3000 rpm, 5 min) and filtration (220 nm syringe filter). To determine the desorption isotherms, 20 mL of pure SBPW was poured onto the centrifuged solid and shaken for another 28 days. At the end of this time, sampling was performed as before. Concentrations were monitored with ICP-OES. For nickel, an additional series of solutions with concentrations between 10^–9^ and 3 × 10^–3^ M were prepared and treated as detailed above and labeled with ^63^Ni radiotracer of 16.6 kBq. As reference, 20 mL each of the starting solutions was labeled with ^63^Ni tracer and kept until the end of the adsorption experiments. The activity concentration of the tracer was measured by liquid scintillation counting (LSC) using a TriCarb system. 200 μL of the filtered samples and the labeled starting solutions were pipetted to 10 mL of Ultima Gold cocktail and measured for 10 min.

Adsorption parameters such as the partitioning coefficient *K*_d_ (L/kg) and the concentration of the sorbed ion *C*_*sorb*_ (mol/kg), can be calculated as follows:
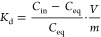
1
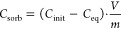
2where *C*_init_ and *C*_eq_ are the initial and equilibrium concentrations of ions of interest (mol/L), respectively, *V* is the volume of the liquid phase (mL), and *m* is the mass of bentonite (g). For the radiotracer measurements, the ratio of the measured activities in the filtered equilibrium solutions (*A*_eq_) and in the initial solutions (*A*_in_) can be used to derive the equivalent concentrations (*C*_eq_).
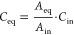
3

The desorption process can be evaluated by the amount of irreversibly adsorbed ion in the solid, which can be calculated using eq 4:
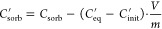
4where *C*′_sorb_ is the concentration of the ion in the solid phase after desorption [mol/kg]; *C*′_eq_ is the concentration of the ion in the liquid phase after desorption [mol/L]; and *C*′_init_ the concentration of the ion in the starting solution before desorption [mol/L].

The concentration of nickel/cobalt ions in the backfilled 20 mL of SBPW can be neglected but can also be estimated by eq 4 below, where the amount of supernatant remaining after centrifugation (about 100 μL, *V*_remain_) can be taken into account.
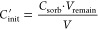
5

#### 2.2.3 Adsorption Modeling

To predict the adsorption isotherm of cobalt ions on B75 bentonite, the 2-site protolysis nonelectrostatic surface complexation and cation exchange (2SPNE SC/CE) model was applied.^19,20^ The model implemented in the PHREEQC Interactive 3.7.3 software^21^ with the ThermoChimie database^22^ was used to calculate adsorption isotherms of montmorillonite in the pore water chemistry conditions of SBPW. Surface complexation on strong and weak sites and cation exchange were taken into account as main adsorption mechanisms.^19^

### 2.3 Design of the Experimental Cells

**Figure 1:**
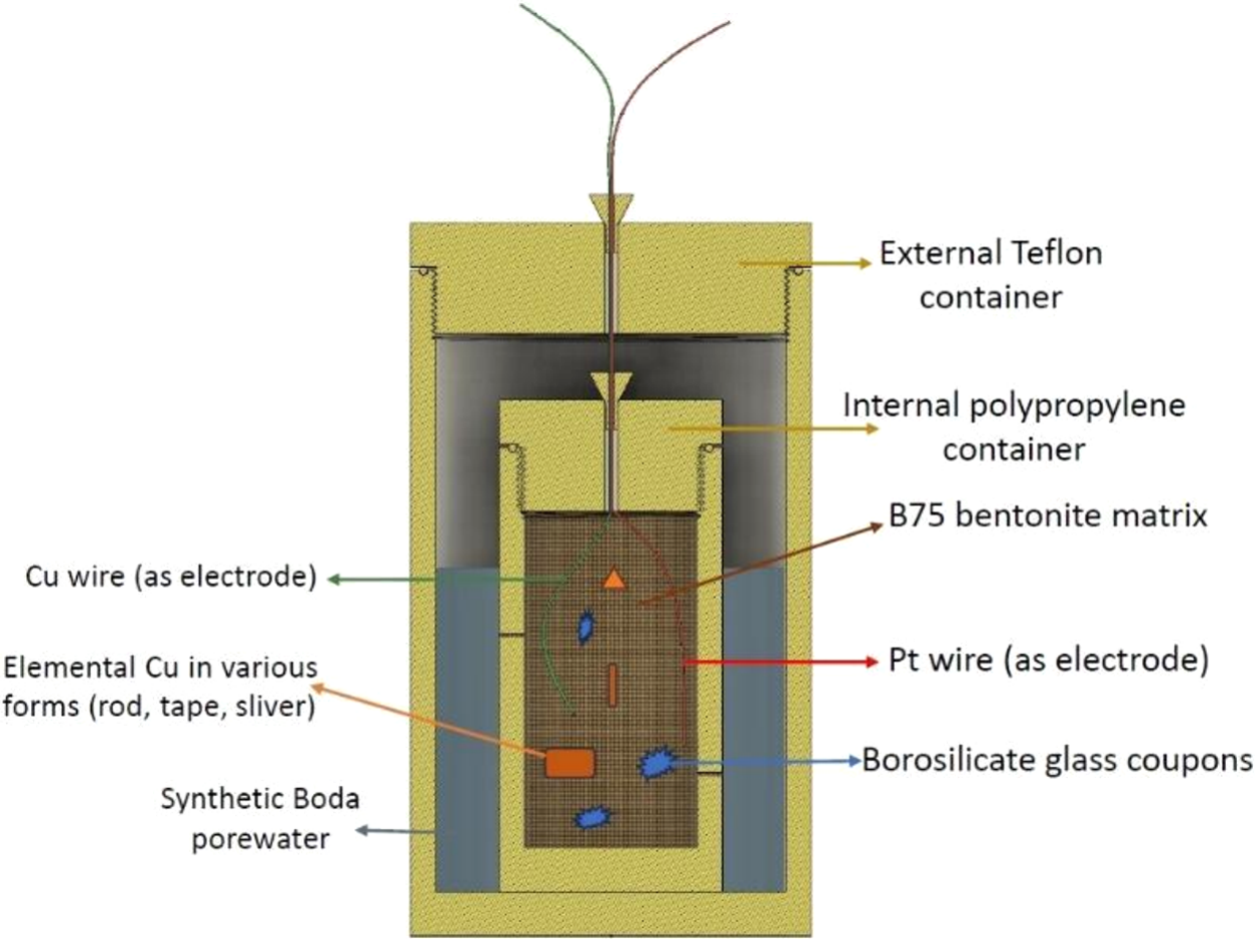
Experimental setup for the glass/copper/bentonite system.

In order to prepare a glass/copper/bentonite model system, experimental cells were prepared in triplicate. For each cell, conditioned B75 bentonite (155 ± 5 g) was placed in a polypropylene (PP) internal container, where the glass and copper pieces were embedded randomly (Figure 1). Five holes with 0.7 mm diameter were drilled in the side of each PP container to establish contact with the pore water poured into the external Teflon container, ensuring constant saturation. All three cells were sealed to maintain anoxic conditions. A constant temperature of 80 ± 2 °C was imposed during the whole experiment. The corrosion potential was monitored using a Pt reference electrode. The Cu and Pt electrodes were inserted into the internal PP container. The experiments ran for 3, 6, and 9 months, and the sample cells were denoted as BEN 3M, BEN 6M, and BEN 9M, respectively. A sample container that ran without heat treatment for 9 months with the same addition of copper and glass (BEN 9M ref) was used as a reference. Each of the experimental setups was opened for postmortem characterization after the respective duration, i.e., after 3, 6, and 9 months.

### 2.4 Characterization Methods

#### 2.4.1 Density and Porosity of Bentonite

Gas density measurements were applied to monitor possible changes in the bentonite. For this purpose, pellets were prepared from the bentonite interior after taking the B75 samples from experimental setups (3–6–9 months). To make the pellets, bentonite was pulverized in an agate mortar to prevent the larger silicate particles from scratching the stamp. The pellets were prepared with SPECAC KBR 25.011 hydraulic press using a 13 mm diameter stamp under 7 tons of pressure. An untreated bentonite pellet sample (BEN UT) was also prepared and measured for comparison. Density was determined using a Micrometrics Accupic II 1340 gas pycnometer. The measurement was performed at room temperature using 5N pure He gas. From the measurement of the gas volume, the density (ρ) and porosity (ε) values can be determined using the following equations:
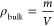
6
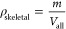
7
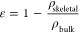
8where *V* is the bulk volume and *V*_all_ is the sum of the volumes of the solid material and closed pores within the material.

#### 2.4.2 Chemical Analysis of Pore Water Solutions

Inductively coupled plasma-optical emission spectroscopy (ICP-OES*)* was used to measure the pore water composition. To prepare the samples, after 3, 6, and 9 months, the bentonite interior from each container was centrifuged, and then the conductivity and pH were determined in the supernatant. The liquid then was analyzed with 1 mg/L yttrium internal standard, diluted 10-fold, and acidified with cc HNO_3_. The measurements were performed using PerkinElmer Avio 200. In the case of Cl^–^ and SO_4_^2–^ ions, IC measurements were carried out using IC-Thermo Scientific Dionex Aquion, with Dionex IonPac CS16 and AS23 analytical columns.

#### 2.4.3 Microanalysis of Copper Pieces

The chemical composition of the copper was checked by scanning electron microscopy and energy-dispersive X-ray spectroscopy (SEM/EDX). Measurements were performed using a Thermo Scientific Scios2 dual-beam microscope equipped with an Oxford X-max*^n^* 20 SDD EDX, at 20 kV accelerating voltage and 1.6 nA sample current. The microscope was operated in low-vacuum operating mode with magnification ranging from 500 to 2000 times.

#### 2.4.4 Characterization of Glass Surfaces

X-ray photoelectron spectroscopy (XPS) was applied to obtain data about the changes in the glass surface composition. XPS measurements were performed using an Escalab Xi^+^ device. Due to the insulating nature of samples, a continuous double charge compensation was applied to prevent electrical charging. The curvature of the glass samples resulted in an uneven charging that was reduced by decreasing the analyzed spot size to 400 μm. The base vacuum used in the measurements was kept at 2 × 10^–10^ mbar, and the XPS measured spot was located at the center of the sputtered square. Each sample was tested in three independent positions. Several assumed minor components were investigated throughout the measurements. Furthermore, note that small amounts of carbon were detected. Still, these data were neglected because carbon, as a nonglass constituent, can only be included in the measurement due to contamination. During the evaluation, the peak intensities were determined from the measured spectra. Decomposition of complex peak shapes with a peak fitting algorithm was applied, where necessary. Component concentration was calculated with sensitivity factors from the ALTHERMO1 library by assuming a homogeneous target.

## 3 Results and Discussion

### 3.1 Retention of Ni^2+^ and Co^2+^ by B75 Bentonite

**Figure 2:**
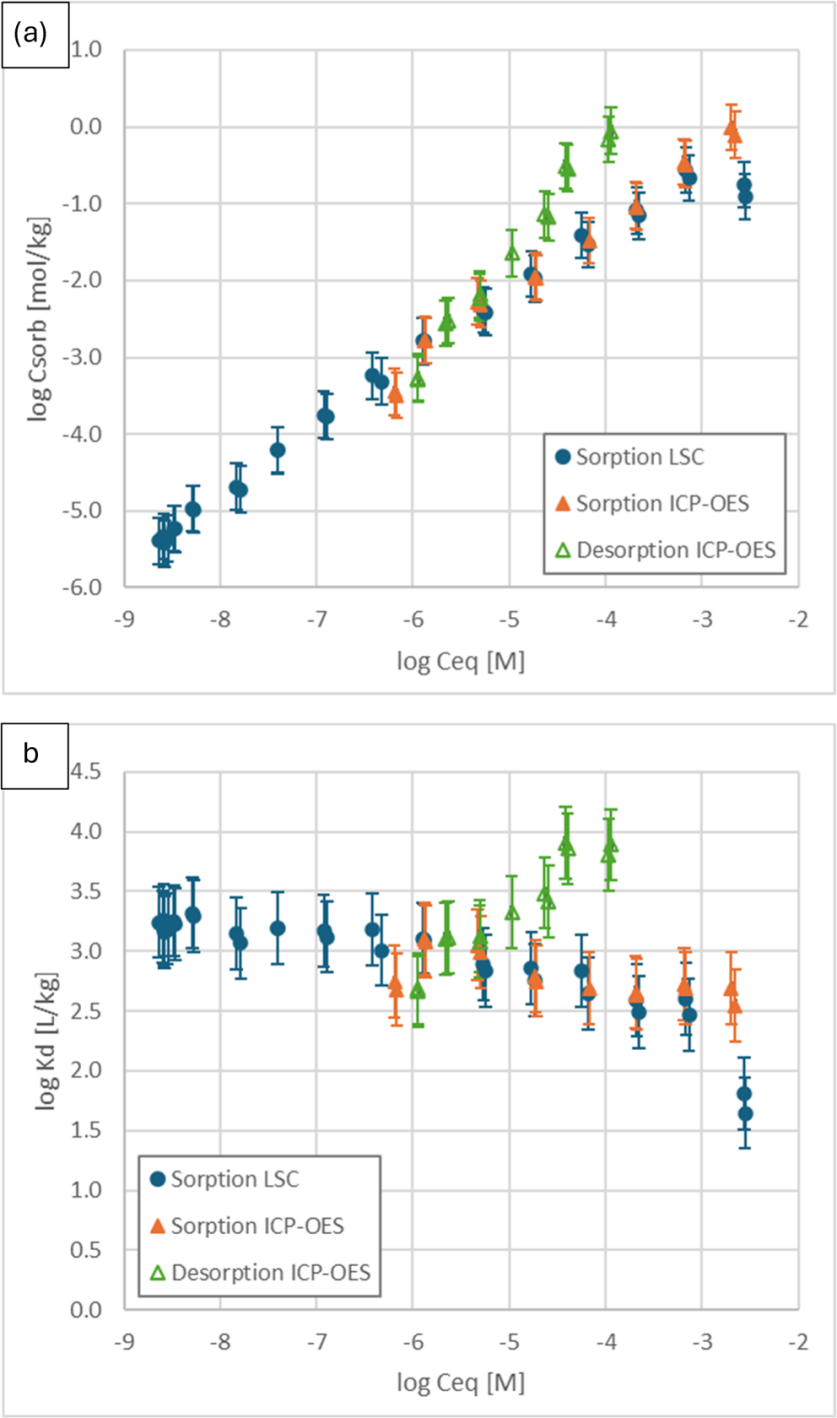
Adsorption and desorption isotherms of nickel on B75 bentonite, expressed as the sorbed amount *C*_sorb_ (a) and the distribution coefficient *K*_d_ (b).

**Figure 3:**
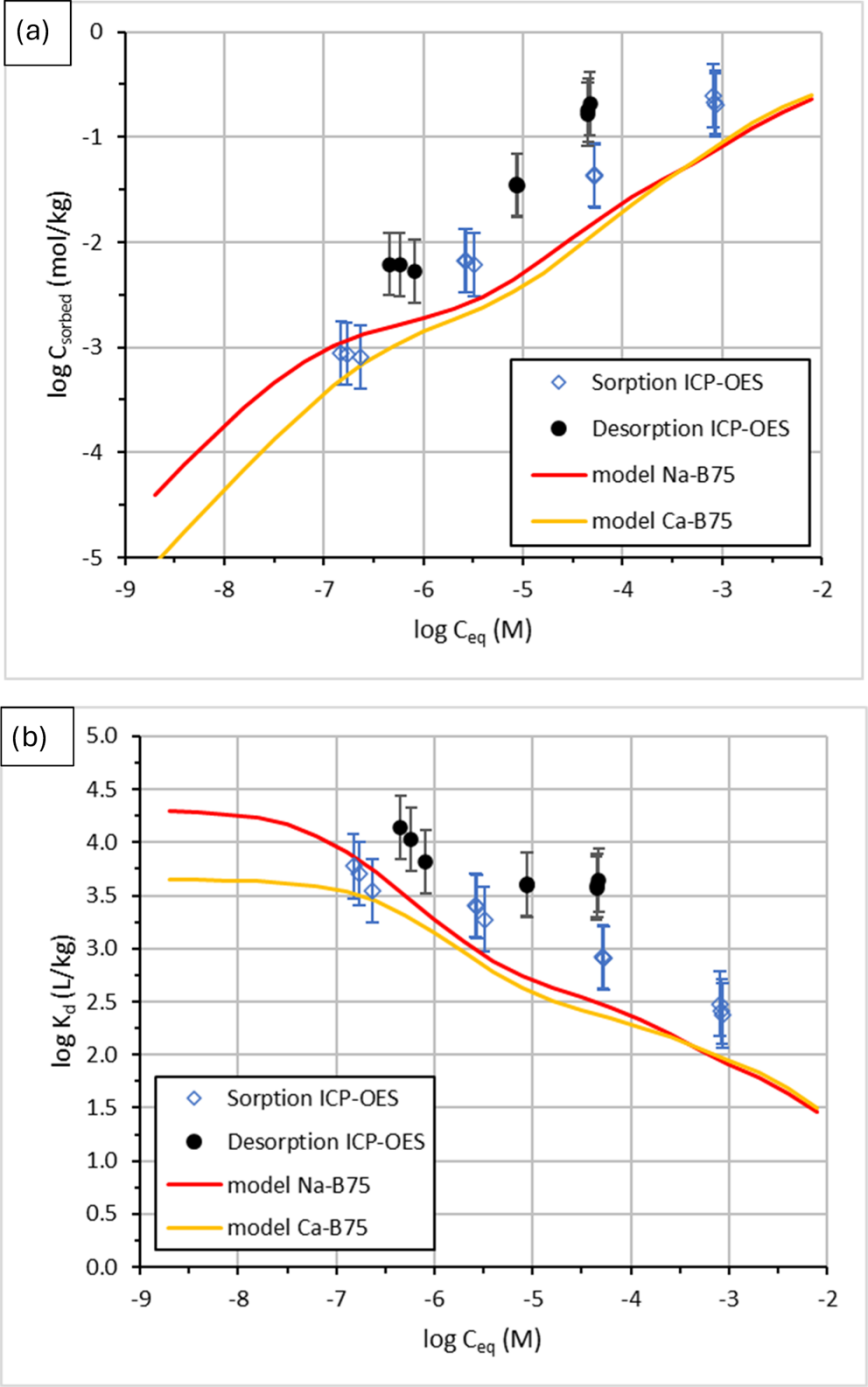
Measured adsorption/desorption isotherms and modeled adsorption isotherms of cobalt on B75 bentonite, expressed as the sorbed amount *C*_sorb_ (a) and the distribution coefficient *K*_d_ (b).

Figures 2 and 3 show the adsorption/desorption isotherms for Ni(II) and Co(II), respectively, illustrated by the distribution coefficient (*K*_d_) and equilibrium concentrations (*C*_eq_). For Ni(II), adsorption isotherms obtained by LSC and ICP-OES are well in line with the 10^–6^–10^–3^ M concentration range. Similar adsorption isotherms were obtained for MX80 and FEBEX bentonites (Na-bentonites).^20,23^ The difference in adsorption and desorption isotherms obtained suggests irreversible sorption of both ions at higher concentrations (above 10^–5^ M), where the difference is higher in the case of the Ni(II) ions than for Co(II) ions. One reason for the irreversible adsorption may be the formation of new phases: at high nickel concentrations (10^–4^–10^–3^ M), the formation of Ni–Al layered double hydroxide around clay particles was reported on pure clay minerals of Illite^12^ and montmorillonite,^24^ and on natural argillaceous rocks as well.^25^ Irreversibility was not only observed in clay-rich rocks: in the case of Israeli soil (Bet Dagan), which is a representative sandy loam, the obtained isotherms demonstrate strong hysteresis, indicating some permanent adsorption and/or precipitation.^26^ X-ray absorption spectroscopy (EXAFS) investigations of Ni(II) sorption on Ca-montmorillonite and Na-Illite also revealed that at higher pH and higher equilibrium concentrations, surface precipitation was responsible for the significant decrease in liquid phase concentrations.^12,27^ For Co(II) ions, the adsorption isotherm calculated by the 2SPNE SC/CE model approximates the experimental data well at the lowest equilibrium concentration (ca. 10^–7^ M). The model curve assuming Ca-montmorillonite as the main clay mineral responsible for Co(II) adsorption provides a good prediction (Figure 3). At higher equilibrium concentrations, the model underestimates the measured sorption values by half an order of magnitude, suggesting that another uptake mechanism occurred that was not taken into account by the model. This is in line with the difference of adsorption and desorption isotherms, suggesting an irreversible uptake explained by the occurrence of surface precipitation at high Co(II) concentrations.

### 3.2 Time Evolution of the Glass/Copper/Bentonite Model System

#### 3.2.1 Corrosion Potential

**Figure 4:**
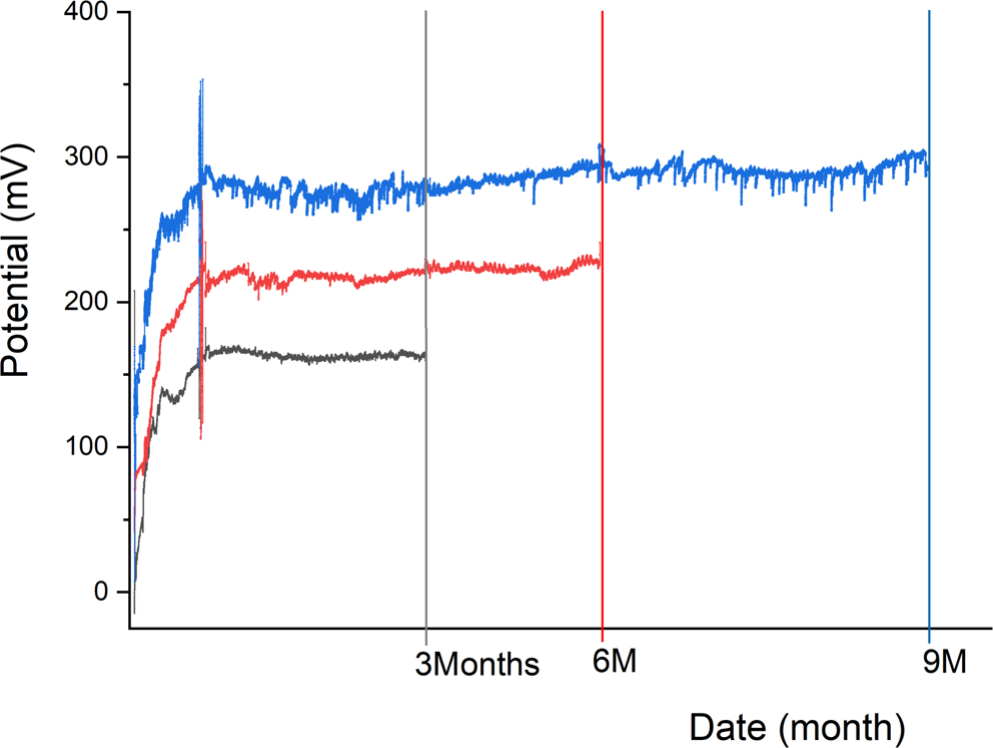
Evolution with time of the measured corrosion potentials for the three cells at 80 °C.

Figure 4 gives the relationship of corrosion potential with months of exposure. Corrosion potential measurement consists of recording the corrosion potential, i.e., the potential difference between the studied material and a reference electrode exp. platinum. If the corrosion potential of a metal increases with time (changes in a positive direction), then protection of the surface occurs, and corrosion slows down. The evolution of the corrosion potential measured on the three glass/copper/bentonite setups (3M, 6M, 9M) follows the same characteristics at all stages of experiments. The corrosion potential measured in the 1st month (for all three setups) shows an increasing trend, then changes to a constant value, and a plateau is formed, which indicates the formation of a passive layer. Film passivation in less than 30 days is notable for all three setups.

Each of the triplicate experimental cells of the glass/copper/bentonite model system was opened after the respective duration of experiment and underwent postmortem characterization. The time evolution of bentonite, pore water, copper, and glass was studied by comparison of samples taken after 3, 6, and 9M and the reference.

#### 3.2.2 Density and Porosity of Bentonite

The results obtained for the untreated bentonite (BEN UT) (Table 2) correlate well with those previously measured by Št’ástka et al. in the Czech Mock-up Josef experiment.^11^ Higher density and lower porosity were characteristic for the saturated and treated samples, with no significant effect on treatment time and temperature.

**Table 2:** Density and Porosity Data for Bentonite Samples after Different Treatment Times

sample	bulk volume [cm^3^]	bulk density [g/cm^3^]	skeletal density [g/cm^3^]	porosity (ε)
BEN UT	2.46	1.79	2.42	0.35
BEN 3M	2.01	2.26	2.66	0.15
BEN 6M	2.06	2.15	2.72	0.21
BEN 9M	2.02	2.19	2.72	0.20
BEN 9M ref	1.98	2.22	2.70	0.18

#### 3.2.3 Chemical Composition of Pore Water Solutions

The elemental and ionic concentrations, along with the pH and conductivity data of the liquid phases taken from the experimental cells after different treatment times, are shown in Table 3. Based on ICP-OES results, a time trend can be observed for the elements dissolved into the pore water. Increasing concentration was detected in the case of B, K, and Na ions. In the case of boron, the increase could be traced back to the leaching of the borosilicate glass, as confirmed by the decreasing amount of boron on the surface of the glass, as observed by XPS. The increase in K^+^ is presumably caused by ion exchange from the bentonite interlayer, whereas the increasing trend obtained for Na^+^ could be explained by ion exchange with Ca^2+^ ions. This correlates well with the parallel decrease in calcium ions in the pore water. Another explanation for the change in calcium concentration is the effect of temperature. As the temperature rose, the pH of the pore water decreased, and as the solubility of calcite (calcium carbonate) also decreased, precipitation occurred, as confirmed by the decrease in the Ca^2+^ content of the pore water.^28^

**Table 3:** Elemental and Ion Concentrations, pH, and Conductivity Data for Pore Water Sampled from the Experimental Cells after Different Treatment Times

concentration [mg/L]	B	Ca	K	Mg	Na	Si	Cu	Cl^–^	SO_4_^2–^	pH	cond. (μS/cm)
SBPW	NA^a^	124 ± 5	7.0 ± 0.4	58 ± 2	390 ± 20	5.0 ± 0.3	NA	872 ± 43	192 ± 9	8.03	3390 ± 80
BEN 3M	1.1 ± 0.1	40 ± 2	28 ± 1	33 ± 1	970 ± 40	5.2 ± 0.2	LOD^b^	954 ± 48	309 ± 15	7.05	3350 ± 80
BEN 6M	1.1 ± 0.2	28 ± 2	28 ± 3	26 ± 3	850 ± 30	2.9 ± 0.3	LOD	1003 ± 13	355 ± 11	7.87	3350 ± 80
BEN 9M	3.8 ± 0.2	34 ± 1	54 ± 2	36 ± 1	1640 ± 40	21 ± 3	LOD	1071 ± 31	383 ± 13	7.18	3320 ± 80
BEN 9M ref	0.04 ± 0.01	26 ± 1	55 ± 2	38 ± 1	1720 ± 31	3.5 ± 0.5	LOD	1008 ± 5	327 ± 3	7.78	2990 ± 70

a Not available.

b Limit of detection.

A slightly decreasing amount of Si after 6 months also turns into a distinct increase further in time. Compared with the reference solution free of copper and glass and supported by XPS results on the glass structure, we can conclude that Si and B dissolved from the glass into the pore water. However, the divalent cations (Ca and Mg) show stable values throughout the experiment following an early decline. The concentrations of Cl^–^ and SO_4_^2–^ ions measured by IC showed an increase throughout the course of the experiment. The pore water Cl^–^ concentration gradually increased as pore water saturated the buffer. The sulfate concentration increased substantially in bentonite pore water when compared to the content in the contacting solutions, both saline and fresh. This trend was observed in other experimental investigations^29^ and modeling studies.^30,31^

When the elemental concentrations of the treated samples are compared with those measured in the reference, a significant difference could be observed for B and Si (almost 100 times higher B and 6 times higher Si concentrations than those in the reference), indicating the leaching of these glass components into the pore water.

#### 3.2.4 Surface Characterization of Copper Pieces

SEM/EDX measurements were used to investigate the surface of the copper samples. A summary of the detailed SEM images for the Cu 3M, Cu 6M, and Cu 9M samples in the three physical forms is presented in Table 4. Based on the measurements, the elemental compositions on the surface of the copper samples in the three physical forms of rod, tape, and sliver could be compared. The amount of copper on the surface of all samples decreased with treatment time, and the amount of O and C increased simultaneously. An explanation for this phenomenon is that upon saturation, the canister surface will be covered by a duplex corrosion product layer comprising an inner layer of Cu_2_O and an outer layer of basic Cu(II) salts,^28,32^ most likely either malachite (Cu_2_CO_3_(OH)_2_) or atacamite (CuCl_2_·3Cu(OH)_2_), depending upon the relative concentrations of CO_3_^2–^ and Cl^–^ in the pore water. The present data correlates well with those obtained by Kosec et al.^5^ A difference could be observed between the tape, sliver, and rod shapes, with the rod showing higher C and lower O concentrations. This difference could be attributed to variations in CuO and CuCO_3_ concentration on the surface of the rod compared to different physical forms. Another notable observation is the appearance of S in the Cu samples taken from the experimental cells after 3- and 9-month treatment. S was detected in nearly half of the Cu samples; this finding also showed a correlation with the increased SO_4_^2–^ content in bentonite pore water.

**Table 4:**
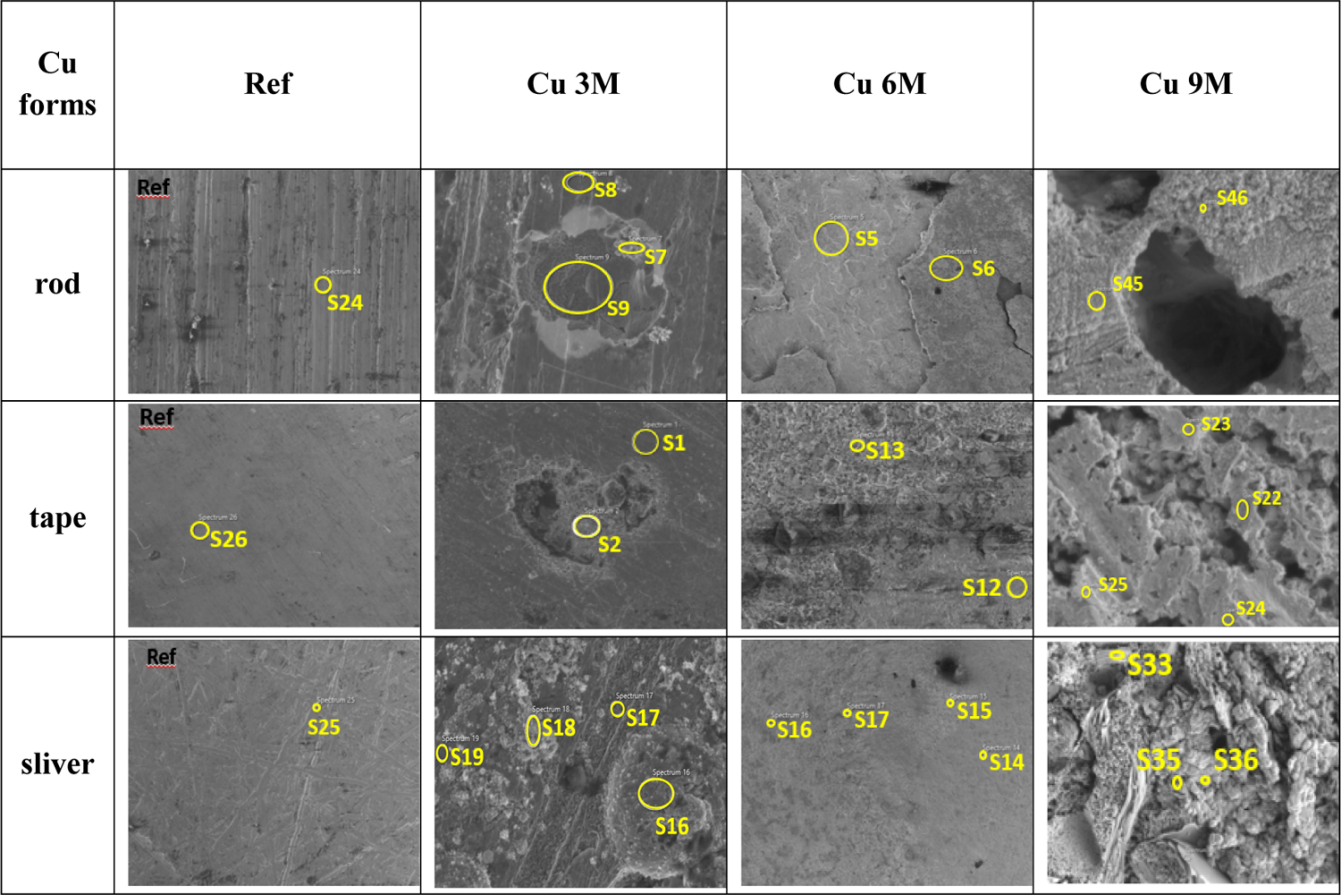
Summary of the SEM/EDX Measurement Point

**Figure 5:**
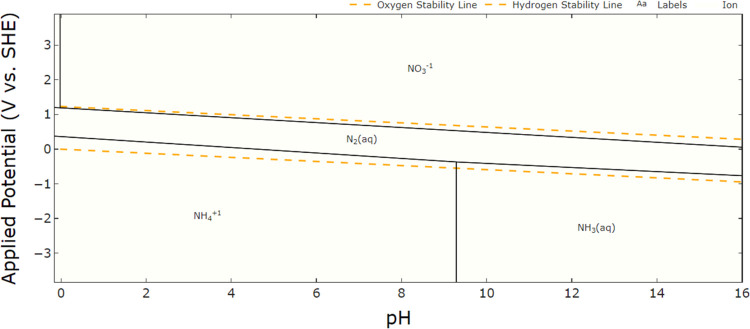
Pourbaix diagram for nitrogen.^34^

Nitrogen detected on the Cu surfaces can be explained by the presence of NH_3_, NH_4_^+^, and NO_3_^–^ complexes. The stability areas of these ligands at 25 °C are shown on the Pourbaix diagram for nitrogen (Figure 5). Nitrogen is present only in trace amounts in SBPW; therefore, most studies have modeled Pourbaix diagrams at unrealistically high N concentrations to investigate the complex formed. This suggests that Cu(NH_3_)^2+^ is formed under our experimental conditions and also ammonia complexes of copper(I) appear important.^33^

According to Pourbaix diagrams published by Muurinen and Lehikoinen,^29^ mostly CuCl_3_^2–^ or CuCl_2_ should have been found under the applied conditions; in contrast, according to our SEM/EDX results, no Cl was detected on the surface of the copper samples. In our system, these composition conditions are not met (approximately 34,000 mg/L Cl^–^ would be necessary), so no such phase detectable by SEM/EDX appeared on Cu surfaces.

Tables 5, 6, and 7 show the results of the SEM-EDX analyses for minerals at the copper surfaces after 3, 6, and 9 months of treatment (for rod (Table 5), for tape (Table 6), and for sliver (Table 7)) based on the points marked in Table 4.

**Table 5:** SEM-EDX Elemental Composition of the Cu Rod Surfaces in the Reference (ref), 3M, 6M, and 9M Samples (the Locations of the Corresponding Spectra Are Given in Table 4)

wt %	Spec 24 ref	Spec 7 Cu 3M	Spec 8 Cu 3M	Spec 9 Cu 3M	Spec 5 Cu 6M	Spec 6 Cu 6M	Spec 45 Cu 9M	Spec 46 Cu 9M
O	0.58	4.44	6.07		12.52	21.14	27.47	27.74
C	3.23	12.01	3.78	3.4	3.31	8.07	7.24	6.86
N							6.58	4.75
Si		1.72	0.46		0.25	1.43		
S		16.22	3.74	6.05				
Ti			12.17					
Al						0.4		
Cu	96.19	65.62	73.77	90.56	83.82	68.96	58.71	60.65
total	100.00	100.00	100.00	100.00	100.00	100.00	100.00	100.00

**Table 6:** SEM-EDX Elemental Composition of the Cu Tape Surfaces in the Reference (ref), 3M, 6M, and 9M Samples (the Locations of the Corresponding Spectra Are Given in Table 4)

wt %	Spec 26 ref	Spec 1 Cu 3M	Spec 2 Cu 3M	Spec 12 Cu 6M	Spec 13 Cu 6M	Spec 22 Cu 9M	Spec 23 Cu 9M	Spec 24 Cu 9M	Spec 25 Cu 9M
O	0.9	7.7	8.98	55.52	43.26	33.26	26.69	26.08	27.62
C	4.8	5.23	6.39	4.90	7.80	8.47	9.10	6.26	10.64
N		1.43	1.5			2.26	4.02	2.24	2.32
Si		0.33	1.96	18.99	8.32	5.93	0.64	0.55	0.80
S		5.48	6.62			3.89	3.71	4.97	3.71
Mg			0.29	2.95	1.33				
Al			0.39	6.82	2.71	1.29			
Cu	94.3	79.83	73.86	10.82	36.57	44.90	55.84	59.90	54.92
total	100.00	100.00	100.00	100.00	100.00	100.00	100.00	100.00	100.00

**Table 7:** SEM-EDX Elemental Composition of the Cu Sliver Surfaces in the Reference (ref), 3M, 6M, and 9M Samples (the Locations of the Corresponding Spectra Are Given in Table 4)

wt %	Spec 25 ref	Spec 16 Cu 3M	Spec 17 Cu 3M	Spec 18 Cu 3M	Spec 19 Cu 3M	Spec 14 Cu 6M	Spec 15 Cu 6M	Spec 33 Cu 9M	Spec 35 Cu 9M	Spec 36 Cu 9M
O	0.64	10.8	7.66	9.36	6.56	17.02	25.52	29.40	65.54	32.24
C	3.84	3.54	4.49	4.56	3.63	3.30	2.65	5.87		
N		2.18	1.37	1.41	0.58			4.26		
Si		0.73		0.63		0.84	6.45	0.74	31.21	1.27
S			4.78	1.3	1.04	3.89	3.71			
Al						0.48	2.81		1.92	
Cl		0.92								
K									0.59	
Mg							1.06			
Cu	95.52	81.48	81.71	82.73	88.18	78.36	61.51	59.73	0.75	66.49
total	100.00	100.00	100.00	100.00	100.00	100.00	100.00	100.00	100.00	100.00

#### 3.2.5 Borosilicate Glass Composition

Based on XPS analysis, the change in the composition of the borosilicate glass structure follows a time trend, an increase in Zr, O, and Ca; a decrease in Si, Ba, Na, Mg, and B, whereas a constant K content (presented in Table 8).

**Table 8:** Calculated Average Surface Concentrations for Glass Composition after 3, 6, and 9 Months

	average surface concentrations [atom %]								
	elements/peaks								
samples	Si 2p	Zr 3d	O 1s	Ba 3d5	Na 1s	Ca 2p3	K 2p	Mg KL1	B 1s
glass 3M	17.63	7.77	67.83	0.3	2.87	1.87	0.5	0.77	0.5
glass 6M	14.96	9.34	69.6	0.21	2.17	2.17	0.46	1.1	0
glass 9M	17.8	11.3	65.7	0.1	2.4	2	0.6	0	0
glass ref	20.5	1.67	60.8	1.5	11.73	0.8	0.47	1.37	2

As previously mentioned, this trend of Si and B in the glass surface correlates with the concentrations measured in the pore water by ICP-OES. As a result, a small amount of Si and B has left the glass structure. Silica gel formation occurred due to the increased surface oxygen concentration when a sufficient amount of hydrolyzed silica was available.^16,35^ After the formation, the dissolution slowed down and the concentration of Si stabilized. It is also important to note the strongly increasing amount of Zr, from which we can conclude that ZrO_2_ is deposited on the glass surface. This observation can be confirmed with previous similar measurements.^36^

## 4 Conclusions

We studied the planned materials (glass–copper–bentonite) from various perspectives to optimize Deep Geological Repositories. One of the key issues addressed was the retention of Ni(II) and Co(II) by B75 bentonite, with the results obtained through adsorption and desorption experiments. Additionally, we explored the interactions among the materials in the glass/copper/bentonite model system under repository conditions.

The difference in adsorption and desorption isotherms obtained suggests irreversible sorption of both ions (Ni(II), Co(II)) at higher concentrations (above 10^–5^ M). In the case of the Ni(II) ions, one reason for the irreversible adsorption may be the formation of new phases: at high nickel concentrations (10^–4^–10^–3^ M), the formation of Ni–Al layered double hydroxide around clay particles. For the Co(II) ions, the model curve assumes Ca-montmorillonite as the main clay mineral responsible for Co(II) adsorption at the lowest equilibrium concentration (ca. 10^–7^ M).

The interactions between the glass–copper–bentonite were studied over a 9-month period. The borosilicate glass was analyzed with XPS, and based on the results, the structural changes could be detected. Were obtained ion migrations and could detect composition replacement in the glass structure; in particular, small amounts of Na leach into the pore water and Zr deposing onto the glass surface; it did not experience any significant release of the structural elements like B and Si. This confirms that the chemical composition of the glass could be suitable for stabilizing radionuclides; their structural integrity does not hurt during the experiments, which predicts a stable structure from long-term perspectives.

The surface of the copper was examined by SEM/EDX. The results suggest that the different physically shaped samples are all suitable, compared to previous studies. No trace of copper was found in the pore water, and there is no evidence of structural damage. During the experiments, it was observed that a uniform corrosion process occurred, resulting in rapid passivation of the copper.

Based on the results, we can state that the engineering barriers do not interfere with each other in the tested environment and have maintained their stability throughout the measurement. With copper and borosilicates proving to be suitable container materials and vitrification matrixes, as part of an engineered barrier, this research further highlights the importance of further investigation.

## Data Availability

The authors declare that the data supporting the findings of this study are available within the paper.
